# Modeling the impact of combined use of COVID Alert SA app and vaccination to curb COVID-19 infections in South Africa

**DOI:** 10.1371/journal.pone.0264863

**Published:** 2023-02-03

**Authors:** Musyoka Kinyili, Justin B. Munyakazi, Abdulaziz Y. A. Mukhtar

**Affiliations:** Department of Mathematics and Applied Mathematics, Faculty of Natural Sciences, University of the Western Cape, Bellville, South Africa; Kyushu Daigaku, JAPAN

## Abstract

The unanticipated continued deep-rooted trend of the Severe Acute Respiratory Syndrome Corona-virus-2 the originator pathogen of the COVID-19 persists posing concurrent anxiety globally. More effort is affixed in the scientific arena via continuous investigations in a prolific effort to understand the transmission dynamics and control measures in eradication of the epidemic. Both pharmaceutical and non-pharmaceutical containment measure protocols have been assimilated in this effort. In this study, we develop a modified SEIR deterministic model that factors in alternative-amalgamation of use of COVID Alert SA app and vaccination against the COVID-19 to the Republic of South Africa’s general public in an endeavor to discontinue the chain of spread for the pandemic. We analyze the key properties of the model not limited to positivity, boundedness, and stability. We authenticate the model by fitting it to the Republic of South Africa’s cumulative COVID-19 cases reported data utilizing the Maximum Likelihood Estimation algorithm implemented in fitR package. Sensitivity analysis and simulations for the model reveal that simultaneously-gradually increased implementation of the COVID Alert SA app use and vaccination against COVID-19 to the public substantially accelerate reduction in the plateau number of COVID-19 infections across all the observed vaccine efficacy scenarios. More fundamentally, it is discovered that implementing at least 12% app use (mainly for the susceptible population not vaccinated) with simultaneous vaccination of over 12% of the susceptible population majorly not using the app using a vaccine of at least 50% efficacy would be sufficient in eradicating the pandemic over relatively shorter time span.

## 1 Introduction

The COVID-19 pandemic whose agent initiator is the Severe Acute Respiratory Syndrome-Corona-virus-2 (SARS-CoV-2) [1–5] has persisted posing anxiety and disbelief in the entire globe. The pandemic seems to preempt attempts to complete the second year while escalating following its first report in Wuhan, Hubei province of China in the end of December 2019 [6–9]. The world continues to keep the battling tools against the epidemic animated and improved day-by-day with more effort invested in bringing the disease to an end. The World Health Organization (WHO) had confirmed in record more than 200 million cumulative COVID-19 case infections with over 4.2 million deaths globally by September 15, 2021. By the similar date, the Republic of South Africa via the Department of Health online resource and news portal on COVID-19 [10] had recorded over 2.84 million COVID-19 positive cases with 105,876 actives cases and 84,761 fatalities.

In a prolific effort to defeat the COVID-19 epidemic, many non-pharmaceutical measures adopted globally have greatly aided in containment of the virus spread [11]. This effort oversaw stretching to incorporating information and communication technology (ICT) sector in the fight. This was via development of android applications (apps) working under various requirements and mechanisms [5]. China and several other countries in East and South East Asia were the foremost to adapt this technological pathway finding it successful in simplification of contact-tracing exercise and as a result reducing the spread of SARS-Cov-2 [12]. A study done in [13] gives a detailed analysis of the digital contact-tracing breakthroughs based on their methodologies and technologies in the light of data emerging on international experiences for deployment of digital contact-tracing technology. More works exploring the subject covering the utilization of the digital contact-tracing technique employing different methodological approaches are also done in [14–21]. The technique is based on either data, WiFi or Bluetooth-contact tracing technology. In this belt, the Republic of South Africa joined the link by devising COVID Alert SA app via the Department of Health. Guidelines on the Republic of South Africa’s Department of Health online resource and news portal on COVID-19 [10], state that the COVID Alert SA is a South Africa’s free exposure notification app. It lets people know when they have been into close contact with someone who tested positive for COVID-19. One can start using the app by downloading and installing it on a device that allows Bluetooth connection like a smartphone. The application is completely anonymous for it preserves the user’s confidentiality and security all the time. The app does not even require or store any personal information. In the event of exposure notification, neither personal information is displayed nor disclosed to the person being notified. The usage of the app complies with the socio-technical framework for digital contact tracing annotated in [22].

On the side of the pharmaceutical pathway, rigorous scientific research begun in early 2020 where more than 50 companies commenced investigations to develop vaccines against the SARS-Cov-2 [23]. Since early December 2020, some vaccines such as Pfizer-BioNTech, Moderna, Oxford-AstraZeneca and Johnson and Johnson’s Janssen (J&J) were approved for use in various countries by regulatory bodies [23, 24]. Among these regulatory bodies is the Europe Medicine Agency (EMA), the UK Medicine and Health Products Regulatory Agency (MHRA), and the US Food and Drug Administration (FDA) [25, 26].

Pfizer-BioNTech vaccine received approval for use firstly in UK on December 2, 2020 whereas the Oxford-AstraZeneca and Moderna vaccines getting approval respectively on December 20, 2020 and January 8, 2021 [23]. Mass vaccination campaigns and clinical trials evaluation have shown that Oxford-AstraZeneca, Pfizer-BioNTech, and Moderna vaccines have ability to provide high levels of efficacy against COVID-19 moderate to severe symptoms especially with 2 doses administered 3 to 4 weeks apart [27–30]. However, supply inadequacy and restricted distribution capacity have made the vaccines delivery to be taxing in most countries world-wide with developing countries leading [24, 26]. The procurement of the COVID-19 vaccines in most low- and middle-income countries is being done via COVAX Advance Market Commitment (AMC) Facility which is a global risk-sharing mechanism for the collaborative procurement of the COVID-19 vaccines [26, 31].

In the Republic of South Africa, vaccination against the COVID-19 roll out kicked off on February 18, 2021 with the single-shot J&J vaccine [10]. This marked South Africa as the first country to roll out the J&J vaccine with the reason that the vaccine was confirmed to work better against the most contagious 501Y.V2 (South African COVID-19 variant) unlike the Oxford-AstraZeneca vaccine which the government of the Republic of South Africa had procured earlier [5]. Previous clinical trials report by the US Food and Drug Administration (FDA) [32] had indicated that the J&J vaccine had 57% efficacy against the contagious 501Y.V2 variant. Later on, their report released online on February 24, 2021 showed that the vaccine offered 64% protection level against moderate to severe COVID-19 infections, with the efficacy level rising to 73%, 14 days after vaccination and 82%, 28 days after receiving the dose. Despite cropping up of more COVID-19 variants and many shortcomings associated with the whole vaccination exercise, vaccination continues with deployment of different types of vaccines.

More and more scientific investigations on transmission and control dynamics against the SARS-CoV-2 have been accomplished. Mathematical modeling has been prolific in assembling more insight to such dynamics [5, 33–38]. For instance, a study in [34] utilized a modified SEIR compartmental mathematical model to study the COVID-19 epidemic transmission dynamics. Similar works on the COVID-19 spread dynamics using different devised models were done in [4, 33, 35, 39–47]. A study in [48] modified SEIR mathematical model and used it to study the impacts of social distancing as an intervention strategy against the transmission of the COVID-19 pandemic. The effects of social distancing have also been modeled in the presence of other control measures to contain transmission of SARS-CoV-2 in [7, 35, 49–51]. The work in [25] adapted a compartmental mathematical model to study the COVID-19 spread dynamics with mass vaccination program. Testing against the COVID-19 and Social distancing as part of containment strategies were also factored in. Similar works assimilating vaccination strategy and other mitigation measures have also been done in [23, 24, 26, 31, 52–54]. Studies done in [55–57] use the concept of evolutionary mechanisms to explain different aspects of epidemics such as the COVID-19.

Up to this far, the world is still battling the COVID-19 epidemic, which calls for more scientific investigations. Thus, owing to the fact that implementing the use of the COVID Alert SA app is a gradual process with a challenge that not everyone in South Africa owning a smartphone [5], and the fact that delivery and distribution of anti-COVID vaccines remain a drawback in developing countries [26], this study seeks to develop a deterministic model factoring in the use of COVID Alert SA app and anti-COVID-19 vaccination program as measures to combat the spread of the epidemic. We use the model specifically to assess alternative-amalgamation potential impact of using COVID Alert SA app and vaccination against COVID-19 to the Republic of South Africa’s public in an endeavor to win the battle against the epidemic.

The rest of the paper is structured as follows: We describe and formulate the model in Section 2. Section 3 presents the model properties (positivity of the solution, feasible region, and stability analysis). We devote Section 4 to analysis of results and discussion (model fitting, sensitivity analysis and simulations). Finally, we give the conclusion in Section 5.

## 2 Model description and formulation

We start by giving the description and formulation of the hereby proposed model which factors in the use of the COVID Alert SA app and vaccination against the COVID-19 epidemic in South Africa. We subdivide the time-dependent cumulative human population *N*(*t*) into seven compartments. The classes include the susceptible class (*S*(*t*)), the susceptible class of individuals who are vaccinated (*S*_*V*_(*t*)), the exposed class of individuals who are using the COVID Alert SA app but not vaccinated (*E*_*A*_(*t*)), the exposed class of individuals who are not using the app and initially not vaccinated (*E*(*t*)), the class of individuals tested against the COVID-19 regardless of whether using the app or not (*T*(*t*)), the class of individuals who are infectious (*I*(*t*)) and the class of individuals removed from the disease’s chain of transmission (*R*(*t*)). The model mainly factors in an alternative of either being vaccinated or using the app. We consider this alternative aspect since we firstly noted that implementation of the app use by the general public would take relatively longer time now that not everyone in South Africa owns a smartphone. Secondly, we also noticed that delivery of anti-COVID-19 vaccines is taxing due to the supply inadequacy and restricted capacity of distribution. We emphasize that the exposed class of individuals who are using the COVID Alert SA app (*E*_*A*_(*t*)), accounts for the cumulative number of individuals who receive exposure notifications by the app on their close contact with people who tested positive for COVID-19. This is absolutely in concurrence with the proper functioning of the app as described in Section 1. Thus, the total human population *N*(*t*) for the model is given by

$$N(t)=S\left(t\right)+S_{V}\left(t\right)+E_{A}\left(t\right)+E\left(t\right)+T\left(t\right)+I\left(t\right)+R\left(t\right).$$ (1)

**Fig 1:**
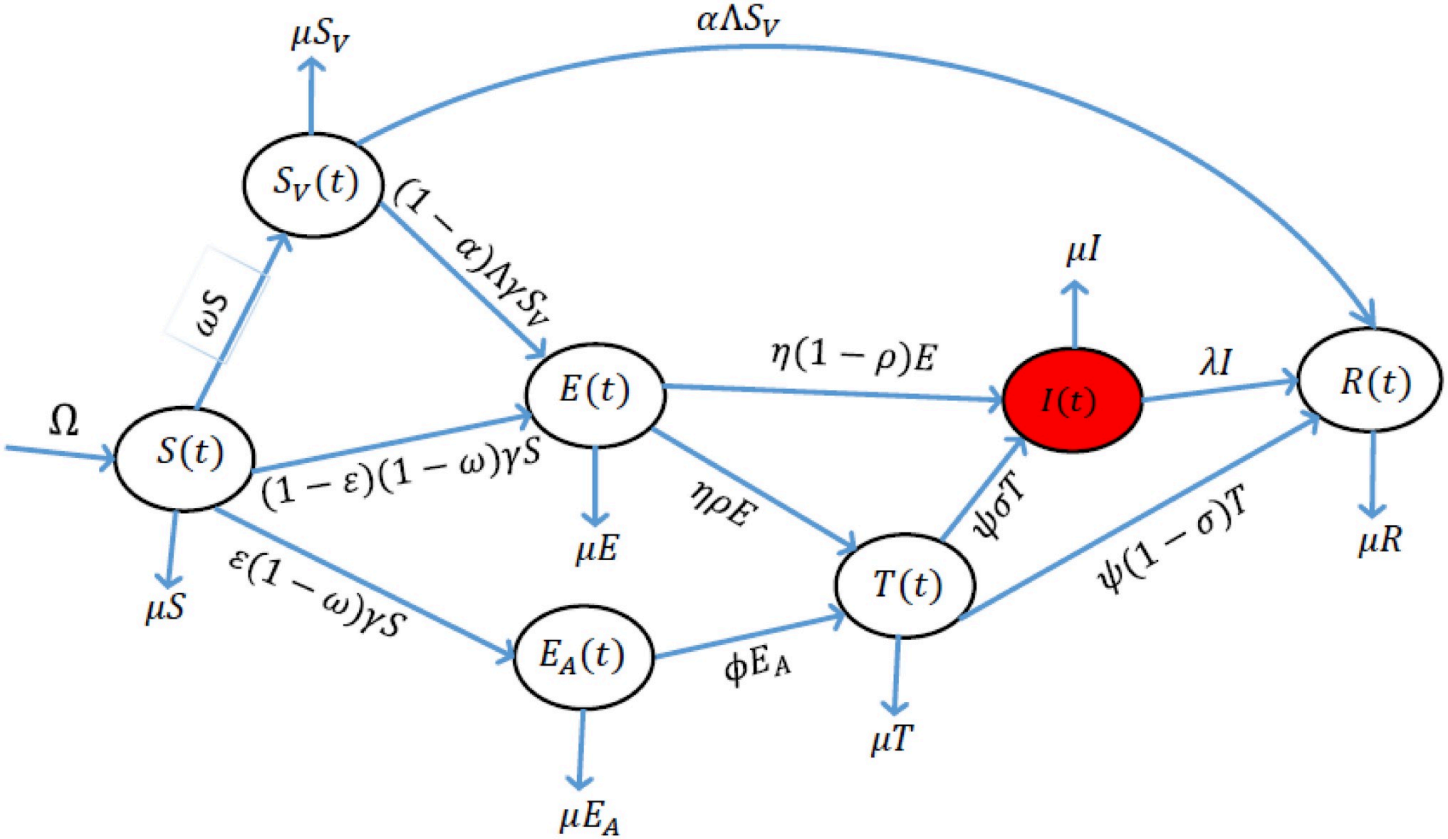
The proposed model incorporating the use of COVID Alert SA app and COVID-19 vaccination.

The flow diagram for the proposed model is shown in Fig 1.

The susceptible individuals *S*(*t*) may either move to the class of individuals who are vaccinated against the COVID-19 (*S*_*V*_(*t*)), or class of individuals who are using the COVID Alert SA app (*E*_*A*_(*t*)) or class of individuals who are not using the app and initially not vaccinated (*E*(*t*)). This consideration is based on the reasons mentioned earlier in this section. The susceptible population gets vaccinated at the rate of *ω*. The vaccinated individuals can get exposed to the disease at the rate of (1 − *α*)Λ following effective contact with the infectious individual(s). This assumption is validated by the facts that the already approved COVID-19 vaccines are not 100% efficacious, and that most vaccines are two-dose prescription in which perhaps there could be high chances of delaying or even missing the second dose within the clinically prescribed time probably due to the challenges associated with the vaccines delivery and thus there are chances on someone being infected even when vaccinated. The model also assumes that individuals who get vaccinated can also be removed from the disease’s chain of transmission (at the rate of *α*Λ) following their highly boosted immunity and wise decision of choosing to fully continue adhering to the laid down mitigation measure protocols against the epidemic as well.

The model factors in the use of the app (at the rate of *ε*) especially for individuals who are not vaccinated (see the reasons stated in this section, paragraph 1). As emphasized before, the exposed individuals (*E*_*A*_(*t*)) account for the total number of people who receive exposure notifications on their close contacts with the individuals who tested positive for COVID-19. This class of individuals can also be termed as cumulative exposure notifications. The model assumes that individuals who are using the COVID Alert SA app sensibly make an immediate decision to go for a test (at the rate of *φ*) in order to confirm their COVID-19 status after receiving exposure notifications on their close contacts with the persons who tested positive for the COVID-19. This is probably because people fear contracting the disease since they have a chance of dying or stigmatization if they contract the disease. If they test positive, they progress to the infectious compartment at the rate of *ψσ*. If their COVID-19 status turns negative, they achieve removal from the disease’s chain of transmission at the rate of *ψ*(1 − *σ*). This assumption is articulated to the logical behavior change of the individuals in strict compliance with the COVID-19 control measure protocols put in place owing to the fact of fear of succumbing to the disease as well as stigmatization.

The susceptible population who are not using the app and are initially not vaccinated, are assumed to be lowly protected and highly prone to the infections hence progress to the class of latent individuals (*E*(*t*)) following unknown exposure (contact) to (with) the infectious person(s). We note that this class may include individuals who were initially vaccinated but got exposed to the virus. The latent individuals can either progress to the tested class (*T*(*t*)) at the rate of *ηρ* or progress directly to the class of infectious individuals at the rate of *η*(1 − *ρ*). These individuals move for a COVID-19 test either by volunteering perhaps because someone is doubting his/her COVID-19 status, or by government manual contact-tracing exercise, or by effort to meet conditions such as traveling abroad. The individuals who test positive to the COVID-19 are assumed to be isolated or quarantined and/or hospitalized immediately. This assures that they do not contribute to further spread of the infections. The class of infectious individuals (*I*(*t*)), collects all the infectious persons including the symptomatic and the asymptomatic ones. We note that, these are the individuals who shed the virus to the individuals who are either using the app or not, and individuals who are either vaccinated or unvaccinated. These individuals collectively get removed from the chain of transmission of the disease at the rate of λ. They include those who have recovered from the COVID-19 infections and individuals who are deceased due to the COVID-19 [26, 58]. They may also include individuals who are fully vaccinated and continue following the COVID-19 protocols. Since the epidemic has been endemic, we considered a recruitment rate denoted by Ω.

The resulting nonlinear system of differential equations for the model is

$$\begin{array}{ccc} \frac{dS}{dt} & = & \Omega -\gamma (1-\omega )S-(\omega +\mu )S, \end{array}$$ (2)

$$\begin{array}{ccc} \frac{dS_{V}}{dt} & = & \omega S-\left(1-\alpha \right)\Lambda \gamma S_{V}-\left(\alpha \Lambda +\mu \right)S_{V}, \end{array}$$ (3)

$$\begin{array}{ccc} \frac{dE_{A}}{dt} & = & \gamma \varepsilon \left(1-\omega \right)S-\left(\phi +\mu \right)E_{A}, \end{array}$$ (4)

$$\begin{array}{ccc} \frac{dE}{dt} & = & \gamma \left(1-\varepsilon \right)\left(1-\omega \right)S+\left(1-\alpha \right)\Lambda \gamma S_{V}-\left(\eta +\mu \right)E, \end{array}$$ (5)

$$\begin{array}{ccc} \frac{dT}{dt} & = & \phi E_{A}+\eta \rho E-\left(\psi +\mu \right)T, \end{array}$$ (6)

$$\begin{array}{ccc} \frac{dI}{dt} & = & \psi \sigma T+\eta (1-\rho )E-(\lambda +\mu )I, \end{array}$$ (7)

$$\begin{array}{ccc} \frac{dR}{dt} & = & \alpha \Lambda S_{V}+\left(1-\sigma \right)\psi T+\lambda I-\mu R. \end{array}$$ (8)

The term *γ* = *βI* defines the force of infections for this model, where *β* is the effective contact rate of the susceptible individuals (regardless of whether vaccinated or not, and whether using the app or not) with the infectious individuals (symptomatic or asymptomatic infectious individuals).

The model’s state variables and the parameters are defined to be positive since the model monitors human population. They are respectively given in Tables 1 and 2. In acknowledgment of other several modeling research works done on COVID-19 dynamics before our work and for concurrence purposes, we extracted some existing parameter values in the literature and fitted the other parameter estimates not in existence with respect to the proposed model. The parameter estimates are given in Table 3.

**Table 1:** State variables description for the model (2)–(8).

Symbol	Description
*S*	Susceptible individuals
*S*_*V*_	Susceptible-vaccinated individuals
*E*_*A*_	Exposed individuals using the COVID Alert SA app (Exposure notifications)
*E*	Latent individuals not using the app
*T*	Tested individuals against the COVID-19 regardless whether using the app or not
*I*	Infectious individuals
*R*	Individuals removed from the disease’s chain of transmission

**Table 2:** Parameter description for the model (2)–(8).

Symbol	Description
Ω	Recruitment rate
*β*	Effective contact rate
*ε*	Measure of the COVID Alert SA app use
*ϕ*	Rate of test on COVID-19 for the app users who receive exposure notifications
*ω*	Measure of the extent of vaccination
*α*	Vaccine efficacy
*α*Λ ((1 − *α*)Λ)	Rate of removal (latency) by the vaccinated individuals
*ρη* ((1 − *ρ*)*η*)	Rate of test (infection) by the exposed individuals not using the app
*σψ* ((1 − *σ*)*ψ*)	Infection (removal) rate by the tested individuals
λ	Removal rate from the chain of disease transmission by the infectious individuals
*μ*	Natural death rate

**Table 3:** Parameter estimates for the model (2)–(8).

Symbol	Value per day	Source
Ω	11244	[49]
*β*	1.0598	[49]
*ε*	(0,1)	Variable
*ϕ*	0.98	Assumed
*ω*	(0,1)	Variable
*α*	(0,1)	Variable
Λ	0.9	Fitted
*η*	0.1961	[25, 31]
*ρ*	0.86	Fitted
*ψ*	0.0695	Fitted
*σ*	0.6	[59]
λ	0.1429	[53]
*μ*	0.0001	[26]

## 3 The model properties

As aforementioned, our model monitors human population and thus it is crucial to analyze its basic properties. This exercise is paramount particularly for preservation of the model’s epidemiological meaningfulness [63]. In line with this key reason, we perform the analysis of model (2)–(8) properties.

### 3.1 Positivity of the solution

In the following Lemma, we prove that the model solution holds out positive for all *t* ≥ 0.

***Lemma 3.1***

Let *S*(0)>0, *S*_*V*_(*t*)>0, *E*_*A*_(0)>0, *E*(0)>0, *T*(0)>0, *I*(0)>0 and *R*(0)>0. Then *S*(*t*)>0, *S*_*V*_(*t*)>0, *E*_*A*_(*t*)>0, *E*(*t*)>0, *T*(*t*)>0, *I*(*t*)>0 and *R*(*t*)>0, ∀ *t* ≥ 0.

***Proof***

We suppose that the solution of the model (2)–(8) is not positive for all *t* ≥ 0. Then there exist a first time $\tilde{t}>0$ such that

$$\tilde{t}=\text{inf}\{t|S\left(t\right)=0or\;S_{V}\left(t\right)=0or\;E_{A}\left(t\right)=0or\;E\left(t\right)=0or\;T\left(t\right)=0or\;I\left(t\right)=0or\;R\left(t\right)=0\}.$$

Now, if $S(\tilde{t})=0$, then $\forall \;t\in (0,\tilde{t})$, *S*(*t*)>0, *S*_*V*_(*t*)>0, *E*_*A*_(*t*)>0, *E*(*t*)>0, *T*(*t*)>0, *I*(*t*)>0 and *R*(*t*)>0, $\frac{dS(\tilde{t})}{dt}<0$. However, from Eq (2) $\frac{dS(\tilde{t})}{dt}=\Omega >0$. This contradicts the initial assumption that $\frac{dS(\tilde{t})}{dt}<0$, clearly depicting that *S*(*t*)>0. Adapting similar argument, it can be proved that *S*_*V*_(*t*)>0, *E*_*A*_(*t*)>0, *E*(*t*)>0, *T*(*t*)>0, *I*(*t*)>0 and *R*(*t*)>0 for all *t* ≥ 0. Hence proved.

### 3.2 The feasible region

We next establish that the model’s solution is bounded and prevail in the positive region for all *t* ≥ 0. We accomplish this by ascertaining that the biological feasible region predefined here as

$$\begin{array}{ccc} F_{R} & = & \{(S,S_{V},E_{A},E,T,I,R)\in R_{+}^{7}:S+S_{V}+E_{A}+E+T+I+R\leq \frac{\Omega }{\mu }\}, \end{array}$$ (9)

is positively invariant and attracting with respect to the model (2)–(8).

Adding the differential equations in the system (2)–(8) we get

$$\begin{array}{ccc} \frac{dN}{dt} & = & \Omega -\mu N. \end{array}$$ (10)

Solving Eq (10) by separation of variables and integration factor techniques we obtain

$$\begin{array}{ccc} N(t) & = & \frac{\Omega }{\mu }(1-e^{-\mu t})+N_{0}e^{-\mu t}, \end{array}$$ (11)

where *N*_0_ = *N*(0).

By comparison theorems on ODEs Eq (11) (see [60]) yields

$$\begin{array}{ccc} \underset{t\to \infty }{\text{lim}}N(t) & = & \frac{\Omega }{\mu }.\;\;\; \end{array}$$ (12)

Basically, *N*(*t*) is a monotonic increasing function and thus if $N\left(0\right)\leq \frac{\Omega }{\mu }$, then $N\leq \frac{\Omega }{\mu }$, ∀*t* ≥ 0 which infers that *N* is bounded and thus we conclude that the feasible region $F_{R}$ is positively invariant and attracting. Therefore, it is now worthy to consider the dynamics of the model (2)–(8) in $F_{R}$ for all *t* ≥ 0 [5]. Further, we can consider the model as epidemiologically and mathematically well posed in the $F_{R}$ [26, 61].

### 3.3 Stability analysis

We analyze the model’s stability by firstly computing the disease free equilibrium (DFE). The DFE instant stipulates that in the population under investigation, there is no infectious individual [5]. We establish that our model has a unique DFE given by

$$\begin{array}{ccc} E^{0} & = & \left(S^{0},S_{V}^{0},0,0,0,0,R^{0}\right) \end{array}$$ (13)

where

$$\begin{array}{ccc} S^{0} & = & \frac{\Omega }{\omega +\mu },\\ S_{V}^{0} & = & \frac{\Omega \omega }{\left(\omega +\mu )(\alpha \Lambda +\mu \right)},\\ R^{0} & = & \frac{\alpha \Lambda \Omega \omega }{\mu (\omega +\mu )(\alpha \Lambda +\mu )}. \end{array}$$

#### 3.3.1 Local stability

We oversee the model’s DFE local stability by finding the basic reproduction number which we denote by $R_{0}$. The $R_{0}$ accounts for the mean number of secondary infections that emanate from one infectious individual when introduced in a wholly susceptible population [5, 26]. We utilize the next generation operator (NGO) approach in computation of the $R_{0}$ expression [62].

From the model (2)–(8), F and V respectively representing secondary infections and transfer of infections are given by

$$\begin{array}{ccc} F & = & (\begin{array}{cccc} 0 & 0 & 0 & \frac{\beta \varepsilon (1-\omega )\Omega }{\omega +\mu }\\ 0 & 0 & 0 & \frac{\beta (1-\varepsilon )(1-\omega )\Omega }{\omega +\mu }+\frac{\beta (1-\alpha )\Omega \omega }{\left(\omega +\mu )(\alpha \Lambda +\mu \right)}\\ 0 & 0 & 0 & 0\\ 0 & 0 & 0 & 0 \end{array}), \end{array}$$

and

$$\begin{array}{ccc} V & = & (\begin{array}{cccc} \phi +\mu & 0 & 0 & 0\\ 0 & \eta +\mu & 0 & 0\\ -\phi & -\eta \rho & \psi +\mu & 0\\ 0 & -\eta (1-\rho ) & -\psi \sigma & \lambda +\mu \end{array}), \end{array}$$

implying that

$$\begin{array}{ccc} FV^{-1} & = & \left(\begin{array}{cccc} \frac{\phi \psi \sigma }{KMW}\frac{\beta \varepsilon (1-\omega )\Omega }{\omega +\mu } & \frac{\eta (\rho \psi \sigma +(1-\rho )M)}{LMW}\frac{\beta \varepsilon (1-\omega )\Omega }{\omega +\mu } & \frac{\psi \sigma }{MW}\frac{\beta \varepsilon (1-\omega )\Omega }{\omega +\mu } & \frac{\psi \sigma }{W}\frac{\beta \varepsilon (1-\omega )\Omega }{\omega +\mu }\\ \frac{\phi \psi \sigma }{KMW}\ell & \frac{\eta (\rho \psi \sigma +(1-\rho )M)}{LMW}\ell & \frac{\psi \sigma }{MW}\ell & \frac{\psi \sigma }{W}\ell \\ 0 & 0 & 0 & 0\\ 0 & 0 & 0 & 0 \end{array}\right) \end{array}$$

where

$$\begin{array}{ccc} K & = & \phi +\mu ,\\ L & = & \eta +\mu ,\\ M & = & \psi +\mu ,\\ W & = & \lambda +\mu ,\\ \ell & = & \frac{\beta (1-\varepsilon )(1-\omega )\Omega }{\omega +\mu }+\frac{\beta (1-\alpha )\Omega \omega }{\left(\omega +\mu )(\alpha \Lambda +\mu \right)}. \end{array}$$

Thus the model’s basic reproduction number is

$$\begin{array}{ccc} R_{0}\;\;\;\;\;\; & = & \;\;\;\;\;\;\frac{\beta \Omega }{W(\omega +\mu )}\{(\left(1-\varepsilon \right)\left(1-\omega \right)+\frac{(1-\alpha )\omega }{\left(\alpha \Lambda +\mu \right)})\frac{\eta (\rho \psi \sigma +(1-\rho )M)}{LM}+\frac{\phi \psi \sigma \varepsilon (1-\omega )}{KM}\}\\ \;\;\;\;\;\;\;\;\;\;\;\;\;\;\;\;\;\; & & \;\;\;\;\;\;\;\;\;\;\;\;\;\;\;\;\;\;-\frac{\beta \Omega }{W(\omega +\mu )}\sqrt{\frac{\phi \eta \psi \sigma \varepsilon \left(1-\omega \right)(\rho \psi \sigma +(1-\rho )M)}{KLM}\{\left(1-\varepsilon \right)\left(1-\omega \right)+\frac{(1-\alpha )\omega }{\left(\alpha \Lambda +\mu \right)}\}(\frac{1-M}{M})}. \end{array}$$ (14)

We note that our model is locally asymptotically stable if and only if $R_{0}<1$ and unstable if and only if $R_{0}>1$.

#### 3.3.2 Global stability

We prove the model’s DFE global stability adapting the approach used by [62, 63].

***Lemma 3.3.2***

Let the model be expressible in the form, $\frac{dY}{dt}=F\left(Y,Z\right)$, $\frac{dZ}{dt}=G\left(Y,Z\right)$, *G*(**Y**, 0) = 0, where vector **Y** denotes the model’s non-disease classes and vector **Z** is the disease classes. The model’s fixed point $E^{0}=\left(Y^{0},0\right)$ is globally asymptotically stable (g.a.s) if and only if $R_{0}<1$ and satisfies the following conditions.

*C*_1_: $\frac{dY}{dt}=F\left(Y,0\right)$, **Y**^0^ is globally asymptotically stable.
*C*_2_: $G\left(Y,Z\right)=XZ-\tilde{G}\left(Y,Z\right)$, $\tilde{G}\left(Y,Z\right)\geq 0$ for $\left(Y,Z\right)\in R_{+}^{7}$ where $X=\frac{\partial G}{\partial Z}E^{0}$ and $R_{+}^{7}$ is the region where the model makes biological sense.

***Proof***

From the model system (2)–(8), we find that **Y** = (*S*, *S*_*V*_, *R*)^*T*^ and **Z** = (*E*_*A*_, *E*, *T*, *I*)^*T*^. The model’s DFE is $E^{0}=\left(Y^{0},0\right)=(\frac{\Omega }{\omega +\mu },\frac{\Omega \omega }{\left(\omega +\mu )(\alpha \Lambda +\mu \right)},\frac{\alpha \Lambda \Omega \omega }{\mu (\omega +\mu )(\alpha \Lambda +\mu )},0,0,0,0)$ as set up by Eq (13). The point $E^{0}=\left(Y^{0},0\right)$ is g. a. s if $R_{0}<1$, hence

$$\begin{array}{ccc} \frac{dY}{dt} & = & F(Y,0)\\ & = & [\begin{array}{c} \Omega -(\omega +\mu )S\\ \omega S-\left(\alpha \Lambda +\mu \right)S_{V}\\ \alpha \Lambda S_{V}-\mu R\\ 0 \end{array}], \end{array}$$ (15)

thus the condition *C*_1_ is clearly satisfied. For condition *C*_2_ we have,

$$\begin{array}{ccc} XZ & = & \left(\begin{array}{cccc} -(\phi +\mu ) & 0 & 0 & 0\\ 0 & -(\eta +\mu ) & 0 & 0\\ \phi & \eta \rho & -(\psi +\mu ) & 0\\ 0 & \eta (1-\rho ) & \psi \sigma & -(\lambda +\mu ) \end{array})(\begin{array}{c} E_{A}\\ E\\ T\\ I \end{array}\right) \end{array}$$ (16)

and

$$\begin{array}{ccc} G(Y,Z) & = & (\begin{array}{c} \beta \varepsilon \left(1-\omega \right)IS-\left(\phi +\mu \right)E_{A}\\ \beta \left(1-\varepsilon \right)\left(1-\omega \right)IS+\beta \left(1-\alpha \right)\Lambda IS_{V}-\left(\eta +\mu \right)E\\ \phi E_{A}+\eta \rho E-\left(\psi +\mu \right)T\\ \psi \sigma T+\eta (1-\rho )E-(\lambda +\mu )I \end{array}). \end{array}$$ (17)

From the rule that $G\left(Y,Z\right)=XZ-\tilde{G}\left(Y,Z\right)$, then it implies

$$\begin{array}{ccc} \tilde{G}\left(Y,Z\right) & = & (\begin{array}{c} \beta \varepsilon \left(1-\omega \right)I(1-\frac{S}{N})\\ \beta \left(1-\varepsilon \right)\left(1-\omega \right)I(1-\frac{S}{N})+\beta \left(1-\alpha \right)\Lambda I(1-\frac{S_{V}}{N})\\ 0\\ 0 \end{array}). \end{array}$$ (18)

We note that since, 0 ≤ *S*_*V*_ ≤ *S* ≤ *N* then $\tilde{G}\left(Y,Z\right)\geq 0$ hence condition *C*_2_ is satisfied. Based on the proof, we sum up that the model’s DFE is globally asymptotically stable whenever $R_{0}<1$.

## 4 Analysis of results and discussion

### 4.1 Model fitting

**Fig 2:**
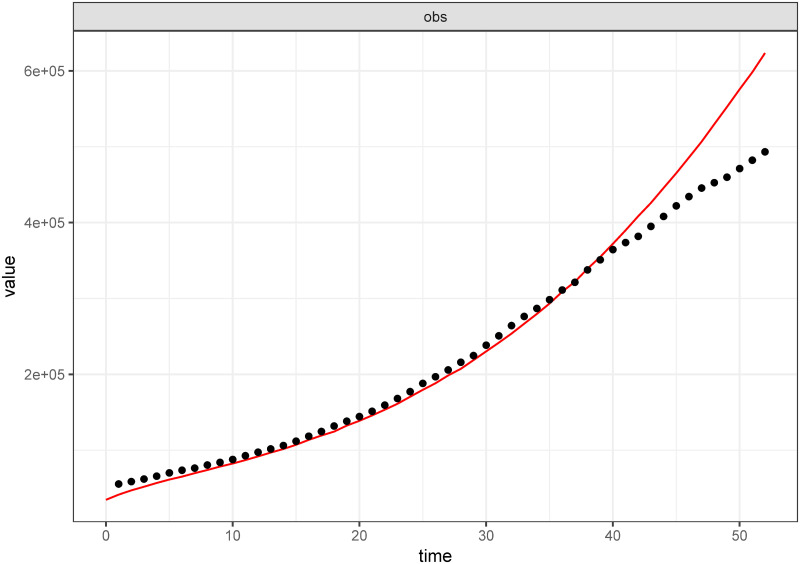
The model (2)–(8) fitting to COVID-19 cumulative positive cases reported in the Republic of South Africa (June 10–July 31, 2020). The black-dotted line denotes the COVID-19 positive cases reported data whereas the red-continuous line represents the model’s best fit. Parameter values used are as given in Table 3 with *ε* = 0.00001, *ω* = 0.00001 and *α* = 0.00001.

For the major purpose of validation of the formulated model for use especially in scenario testing, we fit the model (2)–(8) to real data. We utilize the COVID-19 positive cumulative cases data as reported by the government of the Republic of South Africa [10] in the period between June 10, 2020 and July 31, 2020. We adapt the Maximum Likelihood Estimation (MLE) algorithm implemented in fitR package to achieve the model fitting. The model’s goodness of fit is established by Fig 2.

### 4.2 Sensitivity analysis and simulations

We perform sensitivity analysis and simulations using the fitted model with respect to the parameters of regard. The principal parameters of scrutiny for this research are *ε*, *ω* and *α* denoting measure of use of COVID Alert SA app, measure of extent of vaccination and vaccine efficacy respectively. We define to have poor extent of app use, poor extent of vaccination and poor vaccine efficacy if all the three parameters approach zero whereas perfect app use, perfect vaccination and perfect vaccine efficacy are achieved when all the parameters draw closely to unity thus 0 < *ε* < 1, 0 < *ω* < 1 and 0 < *α* < 1. The sensitivity analysis is principally to oversee the impacts of varying the aforementioned parameters of interest on the basic reproduction number $R_{0}$ while the simulations are majorly for testing of different scenarios [26].

We consider two main sets of vaccine efficacy scenarios namely the general hypothetical vaccine efficacy levels scenario and a specific vaccine efficacy levels scenario. These scenarios are factored in together with concurrently-gradually increasing levels of use of COVID Alert SA app and extents of vaccination. We consider these cases because we noted that implementation of the app use by the general public would take relatively longer time now that not everyone in South Africa owns a smartphone, and the fact that delivery of anti-COVID-19 vaccines is taxing due to the supply inadequacy and restricted capacity of distribution. These reasons among any other shortcomings articulated to the two control measures definitely make the process gradual. We annotate the two scenarios as follows:

- **Scenario One**: ***General hypothetical vaccine efficacy levels***.
  **Owing to the fact that more vaccines are still under development, in this case we consider four levels of vaccine efficacy particularly, *α* = 20%, *α* = 50%, *α* = 70%, and *α* = 90%**.
- **Scenario Two**: ***Specific vaccine efficacy levels***.
  **For this case, we factor in the specific Johnson and Johnson’s Janssen vaccine efficacy trend levels as reported to act against the 501Y.V2 variant (see Section 1). Based on the report, four levels of the J&J vaccine efficacy namely, *α* = 57%, *α* = 64%, *α* = 73%, and *α* = 82% are considered**.

For the sensitivity analysis and the simulations for the itemized scenarios, we use the Parameter values estimates given in Table 3.

#### 4.2.1 Sensitivity analysis

**Fig 3:**
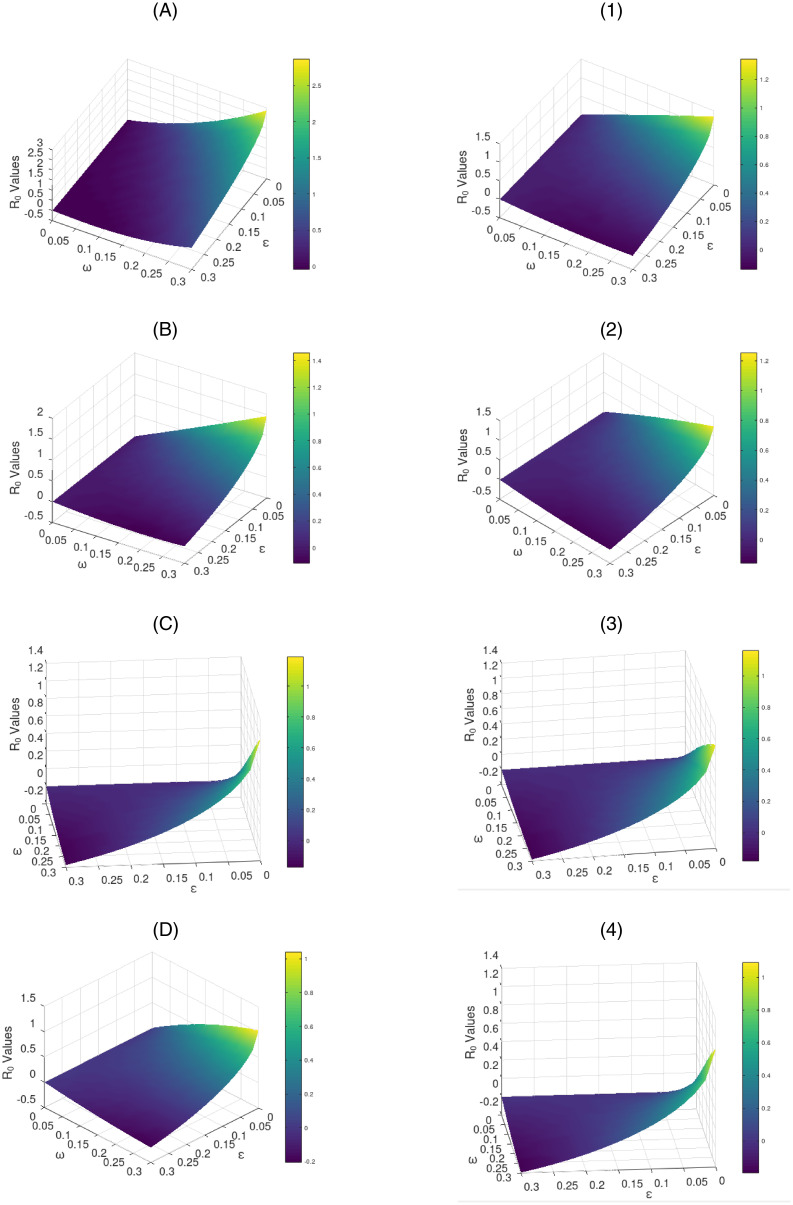
Variation of the $R_{0}$ with *ω* and *ε* for the vaccine efficacy scenarios one (left sub-figures) and two (right sub-figures).

We carry out the sensitivity analysis graphically using Eq (14). Fig 3 with sub-figures A–D and 1–4 respectively for the scenario one and the scenario two, illustrates how the $R_{0}$ varies with *ε* and *ω*. The figure plainly establishes that $R_{0}$ values decrease with increase in the values of the parameters *ε* and *ω* at each level of vaccine efficacy. It distinctively obliques that $R_{0}$ decreases more gently as *ε* and *ω* simultaneously increase gradually at growing levels of vaccine efficacy for each scenario. Observations from the figure suggest that a vaccine of at minimum 50% efficacy would shortly stabilize the disease free equilibrium even with concurrent gradual increase in the measure of app use and the extent of vaccination. This fundamental solitary trend reveals that the simultaneously increased implementation of the COVID Alert SA app use and vaccination against COVID-19 to the public with at least a 50% efficacious vaccine, would greatly reduce the basic reproduction number and thus stabilizing the DFE hence eradicating the pandemic over relatively shorter time.

#### 4.2.2 Simulations

We use the fitted model (2)–(8) to perform simulations for scenarios testing with respect to the parameters of interest. In this case, we confine the simulations to only the model’s disease classes.

**Fig 4:**
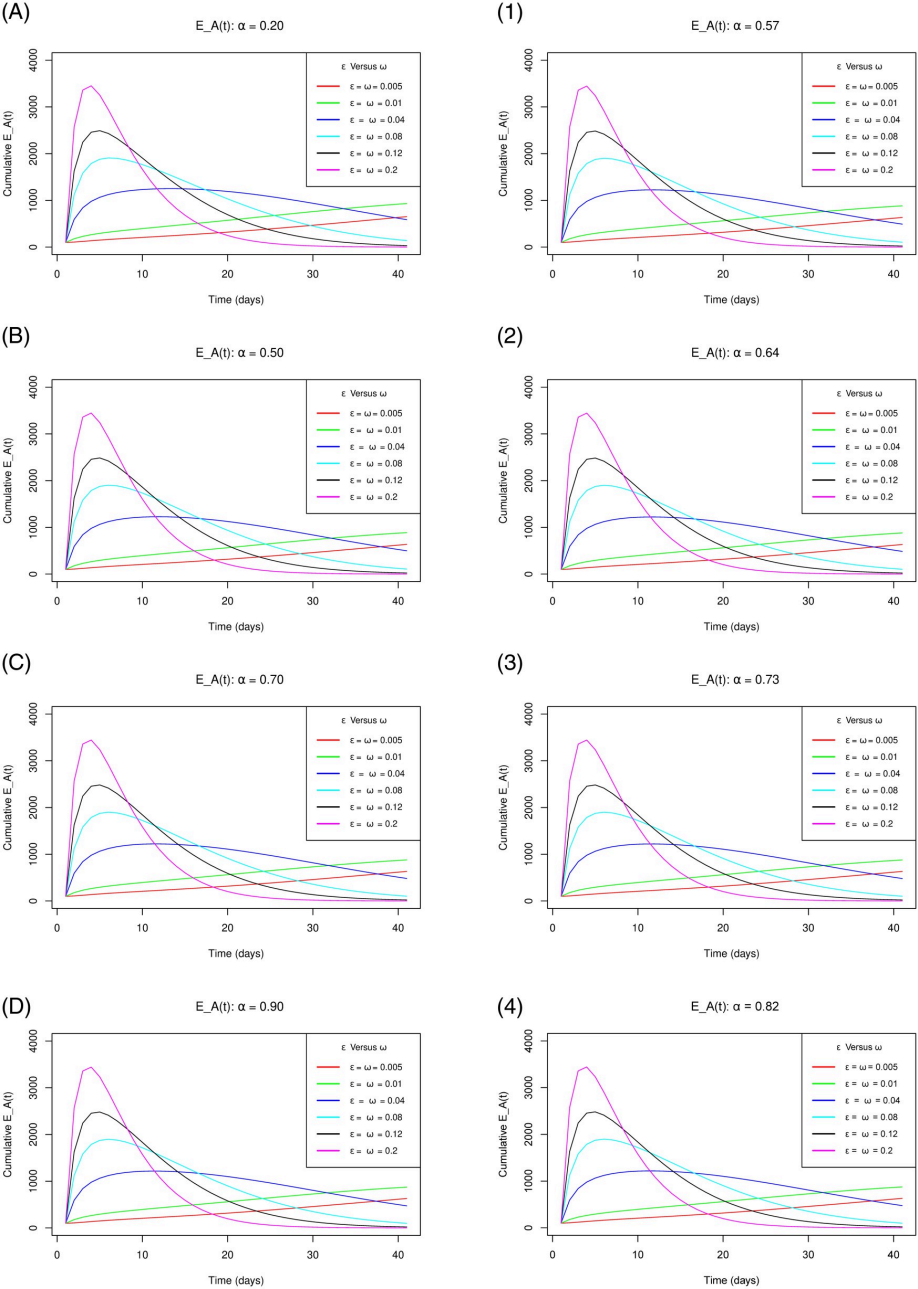
Simulations of the model (2)–(8) depicting the cumulative number of *E*_*A*_(*t*) individuals (cumulative exposure notifications) for the vaccine efficacy scenarios one (left sub-figures) and two (right sub-figures) as *ω* and *ε* simultaneously increase gradually.

Fig 4 with sub-figures A–D and 1–4 respectively for the scenario one and the scenario two, demonstrates how the cumulative number of exposure notifications for individuals using the COVID Alert SA app varies as the parameters *ε* for the measure of use of the app and *ω* for extent of vaccination concurrently increase gradually at increasing levels of vaccine efficacy. It vividly indicates that as the measure of use of the app and the extent of vaccination concurrently increase gradually, the plateau number of the exposure notifications increase gently for both vaccine efficacy scenarios. This solitary trend is absolutely in concurrence with the functioning of the app in exposure notifications to the individuals using the app on their close contacts with persons who tested positive for the COVID-19. The cumulative exposure notifications are anticipated to go up due to the Bluetooth-contact-tracing technology facilitated by the app. A Suggestion from this observation is that the app performs well in contact tracing exercise simplifying it unlike the manual contact tracing that has a high probability of missing several close contacts. These missed close contacts make the COVID-19 positive cases to spike implying that with the use of the app, the pandemic can easily be contained.

**Fig 5:**
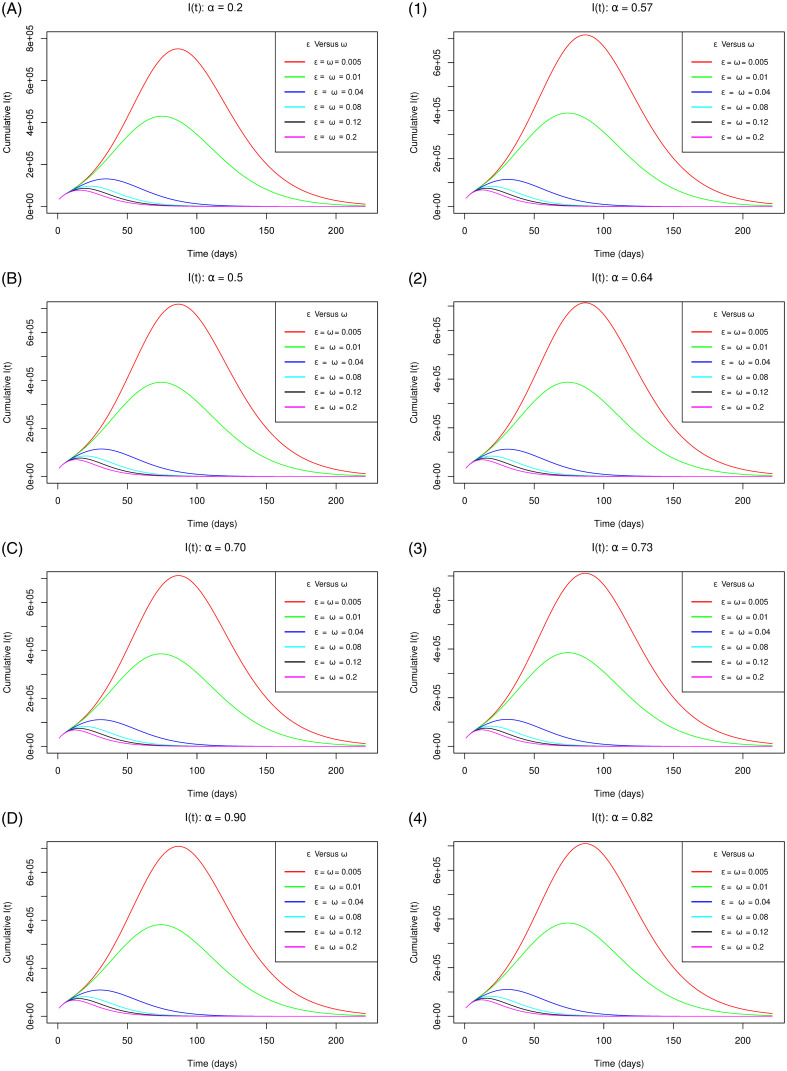
Simulations of the model (2)–(8) depicting the cumulative number of *I*(*t*) individuals (cumulative infections) for the vaccine efficacy scenarios one (left sub-figures) and two (right sub-figures) as *ω* and *ε* simultaneously increase gradually.

Fig 5 with sub-figures A– D and 1–4 respectively for the scenario one and the scenario two establishes, how the cumulative infections vary as the parameters *ε* for the measure of use of the app and *ω* for the extent of vaccination concurrently increase gradually at rising levels of vaccine efficacy. The figure clearly depicts that as the measure of use of the app and the extent of vaccination simultaneously increase gradually, the peak number of the cumulative infections remarkably reduce for both vaccine efficacy scenarios. We clearly observe that synchronously increased use of the app and vaccination accelerates reduction in the COVID-19 infections across all the considered vaccine efficacy levels. This observation is backed up by the reasons that, since people using the app receive exposure notifications when they come into close contact with someone who tested positive for COVID-19, they immediately present themselves for a COVID-19 test. The individuals who test positive are assumed to be isolated or quarantined and/or hospitalized immediately hence do not contribute to further infections. At the same time, contact tracing exercise is simplified. These aspects greatly reduce the chances of unknown exposures and hence leading to reduction in the number of infections. Furthermore, vaccinated individuals gain highly boosted immunity based on the efficacy level of the vaccine which reduces the chances of contracting the COVID-19 infection hence consequentially reducing the number of infections. This solely authenticates that concurrent-gradual implementation of the app use and vaccination would eliminate the pandemic over relatively shorter time.

**Fig 6:**
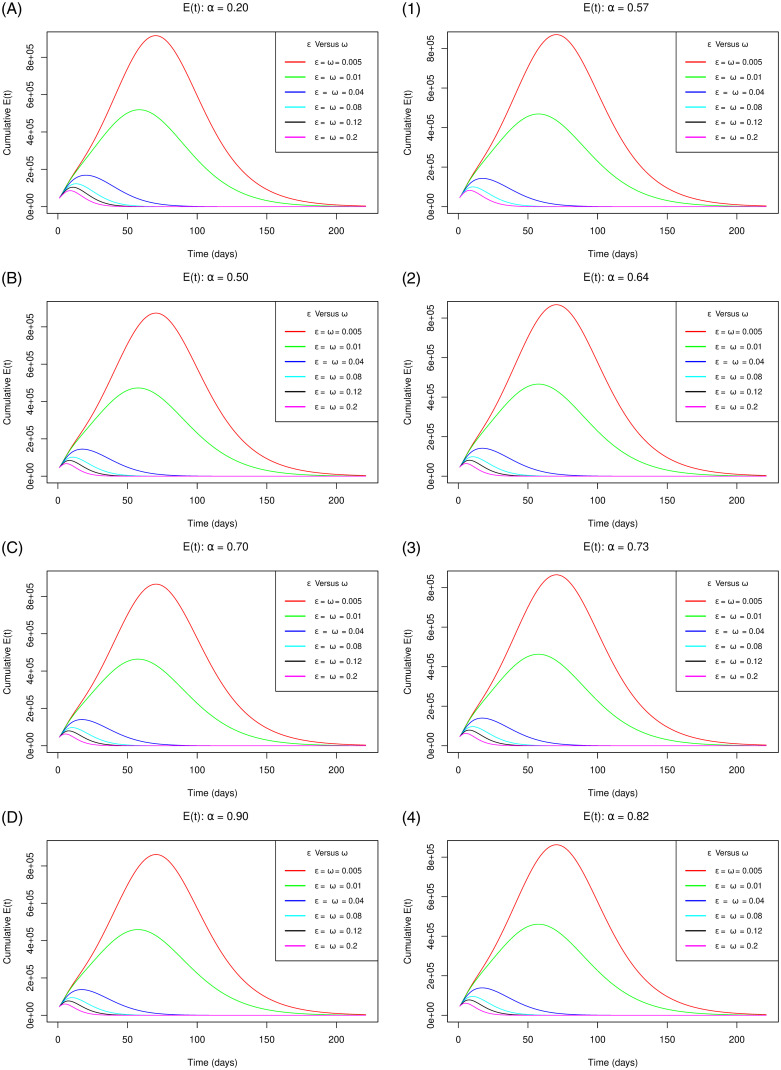
Simulations of the model (2)–(8) depicting the cumulative number of *E*(*t*) individuals (cumulative unknown exposures) for the vaccine efficacy scenarios one (left sub-figures) and two (right sub-figures) as *ω* and *ε* simultaneously increase gradually.

Fig 6 with sub-figures A–D and 1–4 respectively for the scenario one and the scenario two, demonstrates how the cumulative number of exposed individuals not using the app changes as the parameters *ε* for the measure of use of the app and *ω* for extent of vaccination concurrently increase gradually at increasing levels of vaccine efficacy. The figure shows that as the measure of use of the app and the extent of vaccination concurrently increase gradually, it speeds up decrease in the peak number of latent individuals for both of the vaccine efficacy scenarios. This stipulation is supported by the reason that with proper functioning of the app and more people starting using the app, the number of persons not using the app from the susceptible population would decrease. Secondly, the proportion of the individuals not using the app are vaccinated increasingly boosting their immunity hence reducing the chances of being infected. Thirdly, with the concurrent use of the app and vaccination, the number of infections reduce as validated by Fig 5. These reasons would trigger the plateau number of latent individuals to go down consequentially.

**Fig 7:**
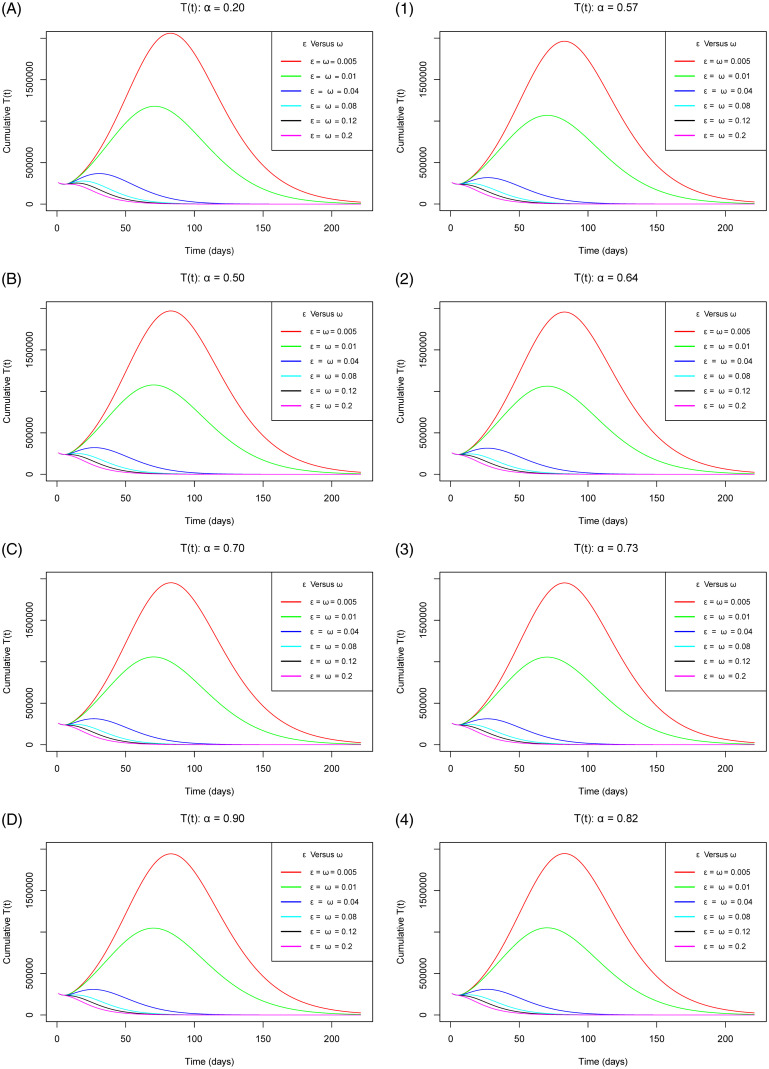
Simulations of the model (2)–(8) depicting the cumulative number of *T*(*t*) individuals (total individuals tested against COVID-19) for the vaccine efficacy scenarios one (left sub-figures) and two (right sub-figures) as *ω* and *ε* simultaneously increase gradually.

Fig 7 with sub-figures A–D and 1–4 respectively for the scenario one and the scenario two, establishes how the cumulative number of tested individuals against COVID-19 (regardless of whether using the app or not) varies as the parameters *ε* and *ω* concurrently increase gradually at increasing levels of vaccine efficacy. The figure exhibits that as the measure of use of the app and the extent of vaccination concurrently increase gradually, the peak number of tested individuals decrease at an accelerated rate for both of the vaccine efficacy scenarios. This trend is backed up by the fact that with the simultaneous use of the app and vaccination, the number of infections and latent cases decrease as validated by Figs 5 and 6 respectively. These reasons would fuel the plateau number of tested individuals to draw low consequentially.

## 5 Conclusions

In this study, we proposed a modified SEIR deterministic mathematical model. The key unique features of the model were the incorporation of use of COVID Alert SA app and vaccination against the COVID-19 to the Republic of South Africa’s general public in a prolific effort to discontinue the chain of spread for the novel COVID-19 epidemic. We performed the analysis of key basic model’s properties ascertaining that the model solution persisted positive and bounded for all non-negative time within a defined invariant region.

We further fitted the model to the reported cumulative COVID-19 positive cases data of the Republic of South Africa. The fitting was implemented in fitR package using the Maximum Likelihood Estimation algorithm. This exercise authenticated the model’s use for simulations.

The model’s sensitivity analysis carried out revealed that simultaneously increased implementation of the COVID Alert SA app use and vaccination against COVID-19 to the public resulted in remarkable decrease in the basic reproduction number. It was established that vaccine of at minimum 50% efficacy would shortly stabilize the disease free equilibrium even with concurrent gradual increase in the measure of app use and the extent of vaccination.

As far as simulations are concerned, it was observed that gradual increased use of the COVID Alert SA app with alternative of vaccination majorly on individuals not using the app, would synchronously simplify the contact tracing exercise and result to high reduction in cumulative infections, as well as decrease in the latent cases. The plateau numbers of infections and latent cases reduced remarkably at an accelerated rate across all the considered levels of vaccine efficacy as the measure of app use and the extent of vaccination simultaneously increased gradually for both of the two aforementioned scenarios. With this solitary tendency, it was fundamentally discovered that implementing approximately 12% app use (mainly for individuals not vaccinated) with simultaneous vaccination of over 12% of the susceptible population majorly not using the app using a vaccine of at least 50% efficacy would suffice in the eradication of the pandemic over relatively shorter time.
